# Author Correction: Modulation of microglial phenotypes by dexmedetomidine through TREM2 reduces neuroinflammation in heatstroke

**DOI:** 10.1038/s41598-021-01761-x

**Published:** 2021-11-09

**Authors:** Ping Li, Tingting Shen, Xue Luo, Ju Yang, Zhen Luo, Yulong Tan, Genlin He, Zeze Wang, Xueting Yu, Ying Wang, Xuesen Yang

**Affiliations:** Department of Tropical Medicine, Army Medical University, Chongqing, China

Correction to: *Scientific Reports* 10.1038/s41598-021-92906-5, published online 25 June 2021

The original version of this Article contained an error in Figure 1, where the representative higher magnification pathological image of brain sections in the “Control” group in panel B was mistakenly taken from the same original image file in the “Heat + DEX” group.

The original Figure 1 and accompanying legend appear below.

**Figure 1:**
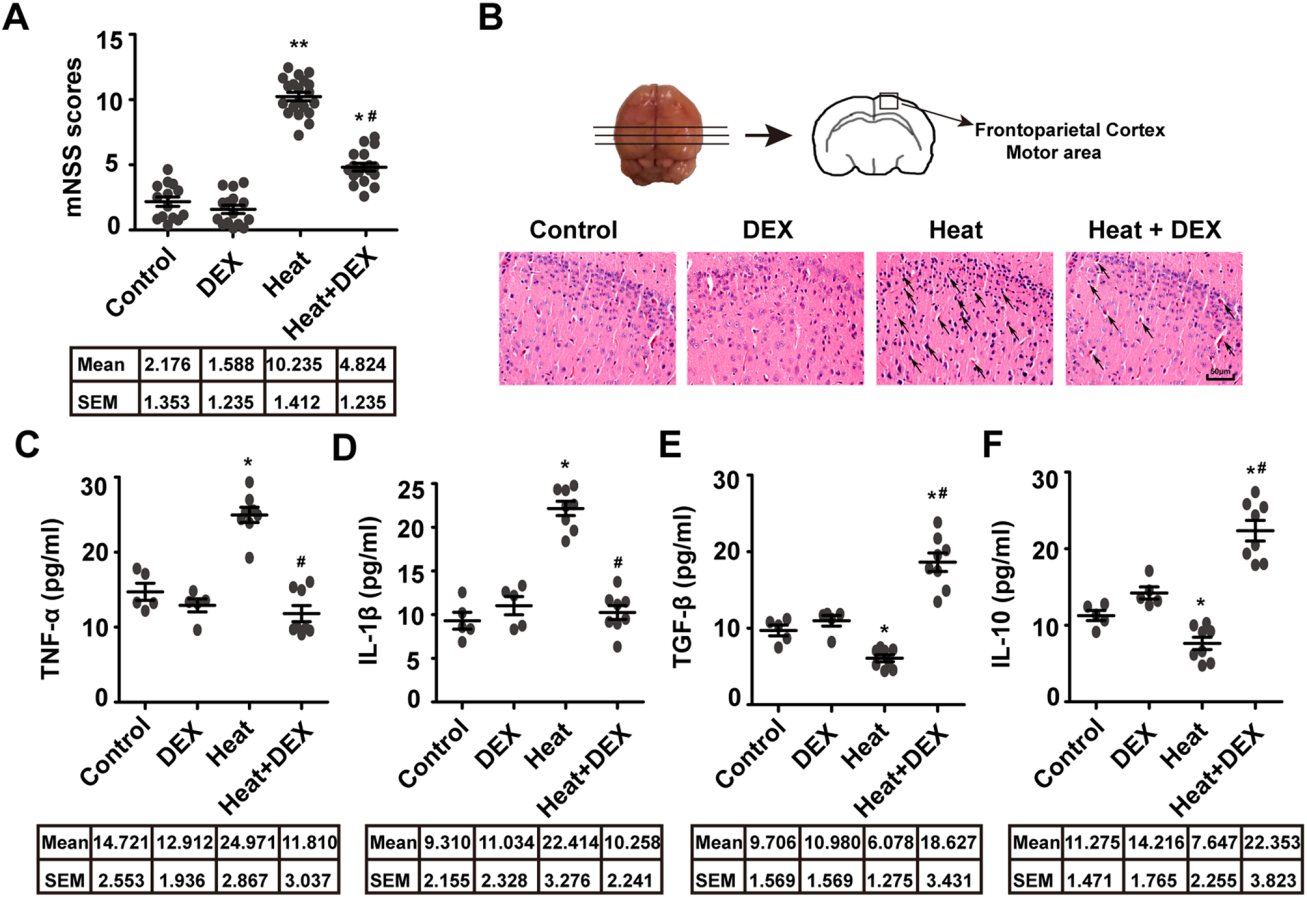
DEX reduces the nerve damage and neuroinflammation caused by heatstroke. ICR mice were exposed to an ambient temperature of 41 ± 0.5 °C until Tc > 42.7 °C (heatstroke onset), and then injected intraperitoneally with 25 µg/kg DEX (Heat + DEX group) or 0.9% saline (Heat group), followed by a 12-h recovery at an ambient temperature of 23 ± 1 °C. (**A**) The neurological function of mice was evaluated by the modified neurological severity score (mNSS). (**B**) Representative pathological pictures of brain sections stained with haematoxylin–eosin (HE) at a magnification of X-200. (**C**–**F**) Cytokine expression levels in the cerebral cortex were assessed by ELISA. The data are shown as the mean ± SEM of three independent experiments. Statistical analysis: two-way ANOVA. *p < 0.05, **p < 0.001 vs. Control. #*p* < 0.05 vs. Heat.

The original Article has been corrected.

